# AN INTERESTING YET UNCOMMON PRESENTATION OF SENSORINEURAL HEARING LOSS DUE TO HYPOTHYROIDISM

**DOI:** 10.51894/001c.123070

**Published:** 2024-08-30

**Authors:** Suhitha Bysani

**Affiliations:** 1 Transitional Medicine Detroit Medical Center Sinai-Grace Hospital

## 71

### INTRODUCTION

Thyroid hormone is responsible for the development and function of the cochlea through fast-acting potassium channels. Thyroid-stimulating hormone (TSH) receptors also regulate the myelination of the cochleovestibular nerve and the integrity of the cochlear cytoskeleton. It has been suggested that there is a direct signal pathway from the thyroid to the inner ear spiral ganglion cells and inner and outer hair cells. Hypothyroidism disrupts the morphology of the outer hair cell and reduces the cochlear microtubules via decreased fibroblast growth factor (FGF) and cofilin gene expression. This may provide a link between thyroid dysfunction and sensorineural hearing loss.

### CASE DESCRIPTION

A 64-year-old female, with a past medical history of hypertension and hypothyroidism secondary to radioactive iodine ablation, presented after a fall. Home medications included levothyroxine 175 mcg daily; however, she was nonadherent with her medication. A thyroid profile obtained in 2022, one year preceding this hospital admission, showed TSH 54.80 micro-IU/mL and free T4 0.3 ng/dl. A few months later, the patient developed sudden bilateral sensorineural hearing loss that was more pronounced in the left ear. Audiogram revealed moderate to profound mixed hearing loss on the right and moderate mixed hearing loss on the left, with asymmetry on her word recognition score - worse on the right. Marginal improvement in hearing following cerumen disimpaction was observed. During this admission, her thyroid profile demonstrated a TSH level of 44.05 micro-IU/mL, free T4 < 0.25 ng/dl, and free T3 2.3 ng/dl. The patient was resumed on her home dose of levothyroxine. A week later, she reported subjective hearing improvement bilaterally with a repeat TSH of 19.88 micro-IU/mL.

### DISCUSSION

This case demonstrates that although thyroid disease has a high incidence globally with well-known symptoms, it may still present with uncommon manifestations. Studies have linked thyroid dysfunction with both sensorineural and conductive hearing loss. Treating hypothyroidism not only prevents hearing loss but also has the potential for complete or partial hearing restoration. Therefore, it is imperative to include TSH levels in the evaluation of hearing loss with uncertain etiology. Additionally, screening for hearing loss in newly diagnosed hypothyroidism can be considered.

